# Age-related changes and effects of regular low-intensity exercise on gait, balance, and oxidative biomarkers in the spinal cord of Wistar rats

**DOI:** 10.1590/1414-431X20198429

**Published:** 2019-07-15

**Authors:** E.M.S. Silveira, A. Kroth, M.C.Q. Santos, T.C.B. Silva, D. Silveira, A.P.K. Riffel, T. Scheid, M. Trapp, W.A. Partata

**Affiliations:** 1 Departamento de Fisiologia, Instituto de Ciências Básicas da Saúde, Universidade Federal do Rio Grande do Sul, Porto Alegre, RS, Brasil; 2 Área de Ciências da Vida, Universidade do Oeste de Santa Catarina, Joaçaba, SC, Brasil

**Keywords:** Superoxide anion generation, Hydrogen peroxide, Total thiol content, Lipid hydroperoxide levels, Total antioxidant capacity, Superoxide dismutase

## Abstract

The present study aimed to analyze age-related changes to motor coordination, balance, spinal cord oxidative biomarkers in 3-, 6-, 18-, 24-, and 30-month-old rats. The effects of low-intensity exercise on these parameters were also analyzed in 6-, 18-, and 24-month-old rats. Body weight, blood glucose, total cholesterol, and high-density lipoprotein (HDL) cholesterol were assessed for all rats. The soleus muscle weight/body weight ratio was used to estimate skeletal muscle mass loss. Body weight increased until 24 months; only 30-month-old rats exhibited decreased blood glucose and increased total cholesterol and HDL cholesterol. The soleus muscle weight/body weight ratio increased until 18 months, followed by a small decrease in old rats. Exercise did not change any of these parameters. Stride length and step length increased from adult to middle age, but decreased at old age. Stride width increased while the sciatic functional index decreased in old rats. Performance in the balance beam test declined with age. While gait did not change, balance improved after exercise. Aging increased superoxide anion generation, hydrogen peroxide levels, total antioxidant capacity, and superoxide dismutase activity while total thiol decreased and lipid hydroperoxides did not change. Exercise did not significantly change this scenario. Thus, aging increased oxidative stress in the spinal cord, which may be associated with age-induced changes in gait and balance. Regular low-intensity exercise is a good alternative for improving age-induced changes in balance, while beneficial effects on gait and spinal cord oxidative biomarkers cannot be ruled out because of the small number of rats investigated (n=5 or 6/group).

## Introduction

Aging is a complex event characterized by a gradual decline of physiological functions and increased susceptibility to disease (1). Among the various theories that attempt to explain the aging process, disruption of the entire signaling network involving reactive oxygen species (ROS) has received increasing recognition over the past two decades (2).

ROS include singlet oxygen, superoxide radicals, hydroxyl radicals, and hydrogen peroxide (H_2_O_2_), which are generated as part of normal cell metabolism and have both normal physiological and pathophysiological functions (3). Excessive ROS formation can induce oxidative stress, leading to cell damage. Therefore, cells possess antioxidant networks that scavenge excessive ROS. Thiols constitute the majority of total body antioxidants and play a significant role in defense against ROS. The antioxidant defense system also includes specific enzymes. The enzymes used to detoxify ROS include superoxide dismutase (SOD), which converts superoxide radicals into H_2_O_2_ (3).

Aging particularly affects the functioning of the central nervous system (CNS). During aging, the CNS experiences ultrastructural modifications, biochemical deficits, inflammatory changes, and impairment of memory, cognition, and locomotor functions linked to oxidative stress (1). The CNS consists of the brain and spinal cord, which compose a single continuous structure. However, mitochondria of the spinal cord have different bioenergetics than those of the brain (4). According to the authors, spinal cord mitochondria appear to produce more ROS and cause more oxidative damage than do age-matched brain mitochondria. Yonutas et al. (4) also observed that ROS production appears to increase by 200 to 600% in the CNS of old Sprague-Dawley rats compared to young rats, specifically in the cortex and in the cervical, thoracic, and lumbar regions of the spinal cord. However, ROS production in this study was assessed using DCF fluorescence. Probes, such as DCF, react strictly by radical mechanisms, and positive responses indicate that radicals, or metal/peroxide complexes capable of undergoing single electron reactions, are being produced (5). Therefore, it appears important to determine the specific changes in ROS with aging. This knowledge will not only improve the understanding of the effects of aging, but will also increase the possibilities of identifying specific mechanisms involved in the aging process.

However, aging also changes the antioxidant system of the spinal cord. The immunoreactivity of SOD, for example, was found to increase in the spinal cord of aged dogs (6). The levels of total thiol, non-protein thiol, and protein thiol were found significantly decreased in the spinal cord of 24–26-month-old rats relative to 3–4-month-old rats (7). However, the gradual changes that occur to SOD activity and total thiol content of the rat spinal cord remain unknown.

Regular physical activity has beneficial effects on age-induced oxidative changes. Exercise programs appear to trigger activation of ROS removal systems in the CNS of young and old rodents (8,9), with the effects seeming to depend on the type of exercise (8). Nevertheless, the effects of regular low-intensity exercise on ROS formation and antioxidants in the rodent spinal cord remain unknown. Recent research demonstrated that low-intensity exercise improved motor function in patients with early-stage Parkinson's disease, especially akinesia (10).

In order to gain insight into previously raised questions, the purpose of this study was to investigate: 1) age-related changes in motor coordination, balance, and spinal cord oxidative biomarkers in 3-, 6-, 18-, 24-, and 30-month-old rats; and 2) the effects of low-intensity exercise on these parameters in 6-, 18-, and 24-month-old rats.

## Material and Methods

### Experimental animals

All procedures involving animals were approved by the Ethics Committee for Animal Experimentation of the Universidade Federal do Rio Grande do Sul (CEUA-UFRGS #29386), with efforts made to minimize animal suffering and reduce the number of animals used. To characterize the effect of aging and regular low-intensity exercise on spinal cord biomarkers, 47 intact 3-month-old male Wistar rats were obtained from a colony maintained by Universidade Federal do Rio Grande do Sul. In the laboratory, the rats were housed in groups of three per conventional cage, maintained at 22±2°C, exposed to a 12-h light/12-h dark photoperiod and provided free access to food and water. Six (n=6) of the rats were acclimated for one week and killed by decapitation. The other rat groups were maintained under laboratory conditions until the ages of 6 (n=12), 18 (n=12), 24 (n=12), and 30 (n=5) months. All rats were kept under close observation and weighed regularly, including prior to sacrifice. Since some rats died of natural causes, the final number of experimental animals was 12 for 6 months, ten for 18 and 24 months, and three for 30 months.

### Exercise protocol

To characterize the effect of regular low-intensity exercise on gait, balance, and spinal cord oxidative biomarkers, half of the 6-, 18-, and 24-month-old rats were randomly selected for an exercise program (exercise rats) at ages of 3, 15, and 21 months, respectively. Since there were only three 30-month-old rats at 27 months, use of these rats in the exercise program was limited. Non-exercise rats remained sedentary.

Prior to initiating the exercise program, the exercise groups were familiarized with a treadmill for one week, 3 times/week, 30 min/day. Having successfully performed the exercise protocol, the rats began the exercise program. The exercise protocol consisted of running on a treadmill designed for human use (Runner, Brazil), but modified for use by rats, for 30 min/day, 3 times/week, for 12 weeks, as described by Sim et al. (11) with some modifications (12). Each session included a warm-up period of 5 min at 2 m/min, then exercise for 5 min at 5 m/min and 20 min at 8 m/min, while the incline was maintained at 0%.

The 12-week exercise program was executed until ages of 6, 18, and 24 months for rats that started at 3, 15, and 21 months, respectively. Exercise was performed only three times a week to avoid chronic stress, inflammation, or muscle damage, and to allow the liver and muscle to recover glycogen; rats received no stimulation (aversive or appetitive) to motivate them to run (12). The duration of 12 weeks was chosen because long-term treadmill exercise was previously shown to be beneficial for the spinal cord (9). The treadmill was thoroughly cleaned between each session.

All rats were sacrificed at the same time of day (beginning at 8:00 am), without fasting. The animals were classified as young adult (3 months), adult (6 months), middle-aged (18 months), young elderly (24 months), and old (30 months) (13,14). These ages were chosen because ages of 6, 18, 24, and 30 months correspond to human ages of 18, 45, 60, and 75 years, respectively (15).

### Functional assessment

To assess sensorimotor deficits, two standard behavioral tests were employed to evaluate motor coordination and balance of rats at defined ages throughout their lifespan: the footprint test and the balance beam test. The footprint test was used to assess stride and step lengths, stride width, and the sciatic functional index (SFI). The balance beam test was used to test the ability to maintain balance while traversing a narrow beam to reach a safe platform.

The footprint test was employed as described by Bloom et al. (16) (to assess stride, step lengths, and stride width) and by de Medinaceli et al. (17) (to assess SFI). Briefly, rats were habituated to the testing apparatus one day prior to starting experimental tests. The apparatus was composed of an open field illuminated by a light (60 W) in which a runway (8.5cm wide, 100cm long, borders 18.5cm high) was arranged leading into a dark wooden box (18×16×18 cm). For testing, the fore paws of the rat being tested were pressed on a red inkpad to dye it, and the hind paws were dyed black. The rat was then allowed to walk across a sheet of paper lining through the illuminated open field leading to the dark box, thus leaving their footprints.

Stride length was defined as the distance between the center of a hind paw strike and the center of the next contact of the same paw on the runway. Step length was defined as the distance between a hind paw strike and the strike of the opposite hind paw. No distinction was made between the left and right measurements for stride and step lengths. Stride width was defined as the distance from the center of a left hind paw strike and the center of the right hind paw strike.

Rat footprints were used to determine the following measurements for the SFI: 1) distance from heel to the third toe (print length, PL); 2) distance from the first to the fifth toe (toe spread, TS); and 3) distance from the second to the fourth toe (intermediate toe spread, ITS). These three measurements were obtained from the left (L) and right (R) paws. Several prints of each foot were obtained on each track, but only three prints of each foot were used to determine the mean measurement for a side. The SFI was calculated using these measurements as: SFI = –38.3 (LPL–RPL) / RPL + 109.5 (LTS–RTS) / RTS +13.3 (LITS – RITS) / RITS – 8.8. The result was considered an index of the functional condition of the sciatic nerve, with zero (±11) representing normal function, and about 100 representing the loss of function.

For gait analysis, the mean value for each variable was calculated for each rat, which was then used to calculate a group mean. All functional measurements were measured manually by two researchers, one of which was blinded to the experimental groupings.

The balance beam test was used as described by Prasad and Muralidhara (18) with minor modifications (19). Briefly, rats were habituated to the testing apparatus one day prior to starting experimental tests. Animals were trained to traverse a 150-cm long narrow wooden beam placed horizontally 60 cm above the floor. The beam extended from a platform at one end to a wooden box (23×23×20 cm) at the other, and was divided into three 50-cm segments (1, 2, and 3). Testing involved recording, by two cameras placed at the platform end of the beam, the number of foot slips off the beam as the rat walked from the platform to the wooden box. The video recordings provided left and right views of the rats as they traversed the beam and provided images for determining the number of foot slips. Scoring ranged from 0 to 8, with a score of 0 for a rat that traversed the beam without foot slips; a score of 1 for a rat that a foot slipped off in the third segment; a score of 2 for a rat that a foot slipped off in the second segment; a score of 3 for a rat that a foot slipped off in the first segment; a score of 4 for a rat that a foot slipped off in the first and second segments; a score of 5 for a rat that a foot slipped off in the first and third segments; a score of 6 for a rat that foot slipped off in the second and third segments; a score of 7 for a rat that a foot slipped off in the first, second, and third segments; and a score of 8 for a rat that did not complete the test.

The balance beam test was also performed to assess the amount of time taken to traverse the beam (in seconds). Score and time were determined for each animal by three researchers, two of which were blinded to the experimental groupings. The mean of the three readings was taken as the score for the animal.

All rats were subjected to a full behavioral test battery prior to sacrifice. The functional tests were conducted at the same time of day (7:00 am) by the same researcher, starting with the balance beam test followed by the footprint test. Two researchers were always present throughout each session, and the behavioral test protocols were highly standardized and maintained over time.

### Sample preparation

Rats were sacrificed by decapitation, and blood, soleus muscle, and the lumbosacral spinal cord were promptly collected. The blood was centrifuged at 1000 *g* for 20 min at 4°C and the plasma used to determine glucose, triglycerides, total cholesterol, and high-density lipoprotein (HDL) cholesterol. Commercially available kits (LABTEST, Brazil) were used for these assays. Triglycerides were assessed because their levels are inversely correlated with executive function in non-demented elderly adults after controlling for age, vascular risk factors, and fiber tract integrity (20).

The soleus muscle and spinal cord were immediately weighed. The ratio of the soleus muscle weight divided by body weight obtained immediately before death was used to estimate the loss of skeletal muscle mass with age as described by Altun et al. (13).

The spinal cord segment was divided transversely into three parts. The same part of each segment always received the same preparation. Two parts were cooled in liquid nitrogen and processed to determine superoxide anion generation (SAG) and H_2_O_2_ levels. A third part was homogenized in 1.15% KCl diluted 1:5 (w/v) containing 1 mmol/L phenylmethylsulfonyl fluoride, centrifuged at 1000 *g* for 20 min at 4°C, and the supernatant was used for assays of total thiols, SOD activity, total antioxidant capacity (TAC), and lipid hydroperoxides levels.

### Antioxidant parameters

Total thiol content was determined as described by Aksenov and Markesbery (21). Briefly, 30 μL of a spinal cord sample was mixed with 1 mL of phosphate/EDTA buffer, pH 7.5, and 5,5′-ditiobis (2-nitrobenzoic) acid (DTNB, 10 mM). Control samples, which did not include DTNB, were run simultaneously. The absorbance was read at 412 nm after 30 min of incubation at room temperature, and the results are reported as mmol/mg tissue.

Total antioxidant capacity was determined with 2,2-azinobis-(3-ethylbenzothiazoline-6-sulfonic acid) radical cation, which, in an acid medium, is decolorized by antioxidants, according to their concentration and antioxidant capacity (22). Results are reported in µmol·eqtrolox^-1^·g tissue^-1^.

SOD activity was measured based on its action to neutralize superoxide radicals to prevent oxidation of adrenaline to adrenochrome, a colored by-product that can be measured at 480 nm. The reaction medium contained glicine buffer (50 mM, pH 11.3) and adrenaline (60 mM, pH 2.0), and the results are reported as units per milligram of protein (23).

### Pro-oxidant parameters

SAG was estimated by using the reduced nitroblue tetrazolium (NBT) method of Wang et al. (24). Briefly, sections of fresh tissue from the lumbosacral spinal cord were mixed with NBT to form formazan as an index of superoxide anion generation. The absorbance of formazan was determined spectrophotometrically at 540 nm. The quantity of NBT reduction = A × V / (T × Wt × Σ × l), where A is the absorbance of blue formazan at 540 nm, V is the volume of the solution, T is the time period (90 min) during which the rings were incubated with NBT, Wt is the blotted wet weight of the spinal cord portion, Σ is the extinction coefficient of blue formazan (i.e., 0.72 L·mmol^-1^·mm^-1^), and l is the length of the light path. Results are reported as reduced NBT pmol·min^-1^·mg tissue^-1^.

To measure H_2_O_2_ levels, horseradish peroxidase (HRPO)-mediated oxidation of phenol red by H_2_O_2_ was used, which leads to the formation of a compound that absorbs at 610 nm. Sections of fresh tissue from the lumbosacral spinal cord were incubated for 30 min at 37°C in 10 mM phosphate buffer (140 mM NaCl and 5 mM dextrose). The supernatants were transferred to tubes with 0.28 mM phenol red and 8.5 U/mL HRPO. After 5 min of incubation, 1 mol/L of NaOH was added and the solution read at 610 nm. The results are reported as μmol H_2_O_2_/g tissue (25).

The lipid hydroperoxides were measured by oxidation of Fe^2+^ by LOOH in an acid medium containing xylenol orange dye, which forms a complex with Fe^3+^, as described by Jiang et al. (26). Results are reported as µmol/g tissue.

### Statistical analysis

The sample size for the present study was based on previous studies using WinPepi software version 9.1 (Professor Joe Abramson, author of WinPepi, http://www.epi-perspectives.com/content/8/1/1), a significance level of 0.05 and 95% power, which resulted in a sample size of eight rats per group. However, some rats died during aging, so the final sample size was 5 or 6 rats per group, except for 30-month-old rats that had a sample size of 3 rats. Data from sedentary rats were compared between different ages to show the effect of aging. Data from exercise rats were compared to sedentary rats to show the effect of regular low-intensity exercise in rats with different ages. For both comparisons, the data were analyzed by two independent researchers, one of which was blinded to the experimental groupings. When there were inconsistencies in the results from the two researchers, a third researcher analyzed the data, and the final result was considered the mean of the results obtained by three researchers. Statistical analyses were performed using SigmaPlot version 11.0 (Systat Software Inc., USA) or Prism 5.03 (GraphPad Software, USA) for Windows. Normal Gaussian distribution of the data was analyzed by Shapiro-Wilk test, while Levene's test was used to analyze homogeneity of variance. To analyze the effect of aging, data for metabolic parameters (blood glucose, triglycerides, total cholesterol, and HDL cholesterol) and from the balance beam test were analyzed by Kruskal-Wallis test followed by Dunn's test. Body weight gain, soleus muscle weight/body weight ratio, footprint analysis, and oxidative biomarker data were analyzed using one-way ANOVA followed by Tukey's *post hoc* test. The Pearson correlation coefficient was used to analyze the correlation between body weight and soleus weight/body weight ratio. To analyze the effect of exercise, data (except for body weight) were analyzed using two-way ANOVA followed by Tukey's *post hoc* test. Data of body weight were analyzed using one-way ANOVA followed by Tukey's *post hoc* test. Tests with P<0.05 were considered statistically significant

## Results

**Figure 1.**
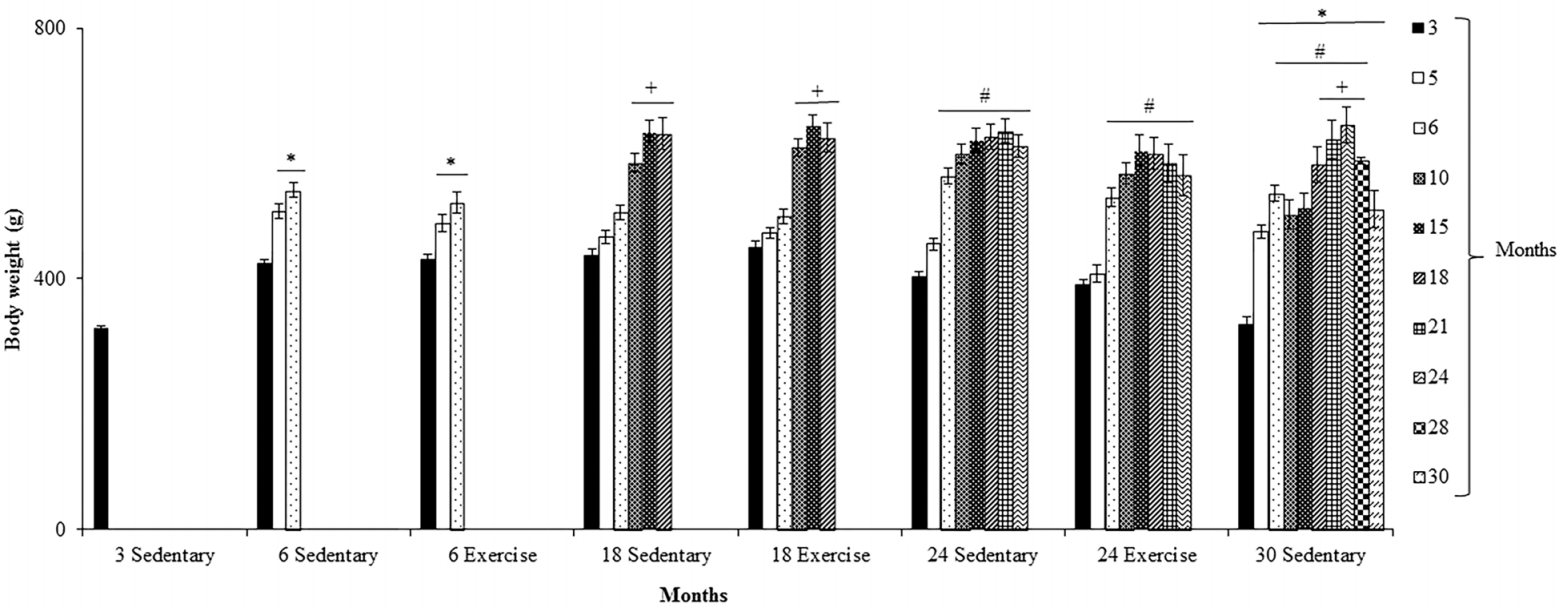
Body weight gain of sedentary Wistar rats 3, 6, 18, 24, and 30 months old and the effects of 12 weeks of treadmill running (Exercise) on this gain in rats 6, 18, and 24 months old. Data are reported as means±SE. *P<0.05 compared to 3-month-old rats; ^+^P<0.05 compared to 3-, 5-, and 6-month-old rats; ^#^P<0.05 compared to 3- and 5-month-old rats (repeated-measures ANOVA followed by Tukey's *post hoc* test).

Since the rats were fed *ad libitum*, they continued to grow and gain weight throughout a considerable portion of their life. Weight gain occurred until 24 months for 24- and 30-month-old rats. Thirty-month-old rats experienced a small decrease (18%) in body weight. These rats also exhibited small decreases in body weight at 10 and 15 months. However, they did not show any change in their behavior or signs of alterations in their physiological functions at these time-points. The exercise program did not induce significant changes in body weight (Figure 1).

**Figure 2.**
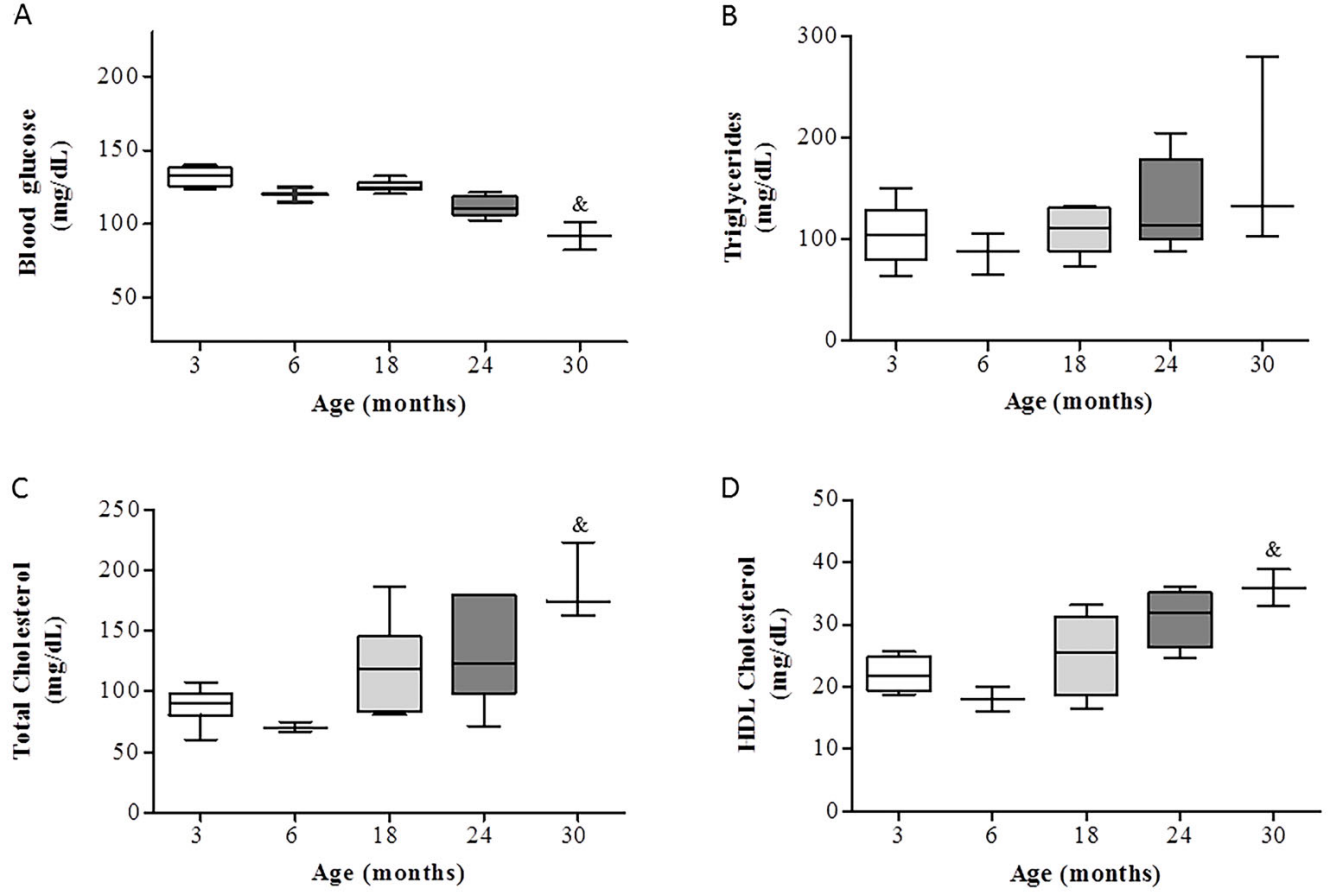
Blood glucose (**A**), triglycerides (**B**), total cholesterol (**C**), and high-density lipoprotein (HDL) cholesterol (**D**) of Wistar rats of different ages. Data are reported as medians and 10–90% interquartile ranges. ^&^P<0.05 compared to all groups (Kruskal-Wallis test followed by Dunn's *post hoc* test).

Blood glucose did not exhibit profound differences among ages, with no significant differences among 3-, 6-, 18-, and 24-month-old rats (Figure 2A). Blood glucose was slightly lower at 30 months, which was significant compared to that at 3 months. There was no significant difference in triglycerides among ages (Figure 2B). Total cholesterol tended to be greater in older rats, but significantly so only at 30 months compared to 3 and 6 months (Figure 2C); a similar result was found for HDL cholesterol (Figure 2D). There were no significant changes in blood glucose, triglycerides, total cholesterol, and HDL cholesterol with exercise.

**Figure 3.**
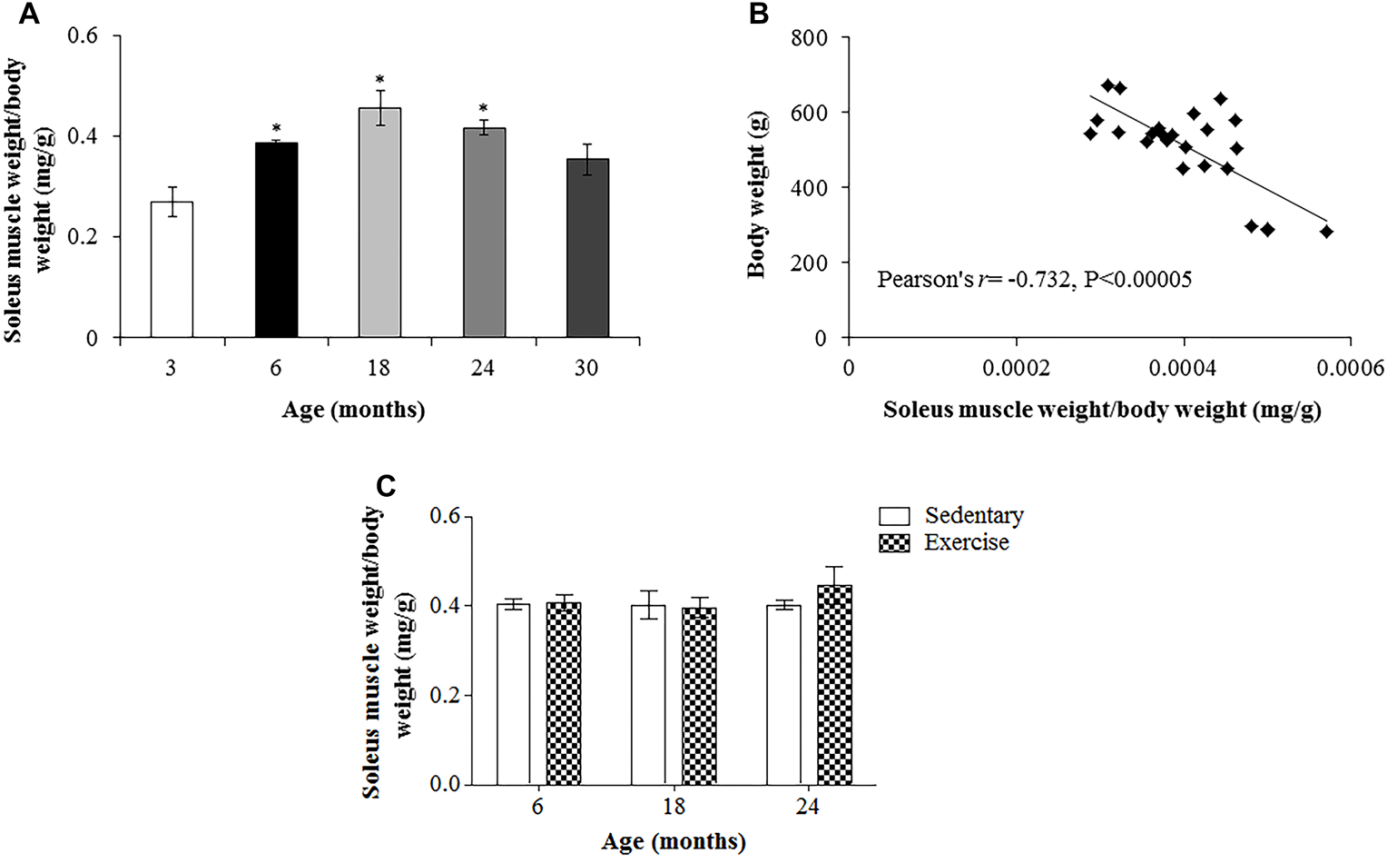
**A**, Ratio of soleus muscle weight (mg) to total body weight (g) of Wistar rats of different ages. **B**, Correlation between total body weight and soleus muscle weight/total body weight ratio. **C**, Effects of 12 weeks of treadmill running on soleus muscle weight (mg)/total body weight (g) ratio of aged rats. Data are reported as means±SE. *P<0.05 compared to 3-month-old rats (repeated-measures (**A**) and two-way ANOVA (**B**) followed by Tukey's *post hoc* test).

The soleus weight (mg)/total body weight (g) ratio differed among ages. This ratio was significantly greater for 6-, 18-, and 24-month-old rats compared to 3-month-old rats (Figure 3A). However, the ratio did not differ significantly between 30 months and 3 months. Although the ratio was approximately 12% lower at 24 months compared to 18 months, and 15% lower at 30 months than at 24 months, the differences were not significant. There was a significant negative correlation between body weight and soleus muscle weight/body weight ratio (r=–0.732, P<0.005; Figure 3B). The exercise program did not induce a significant change in the soleus weight (mg)/total body weight (g) ratio (Figure 3C).

### Functional assessment

**Figure 4.**
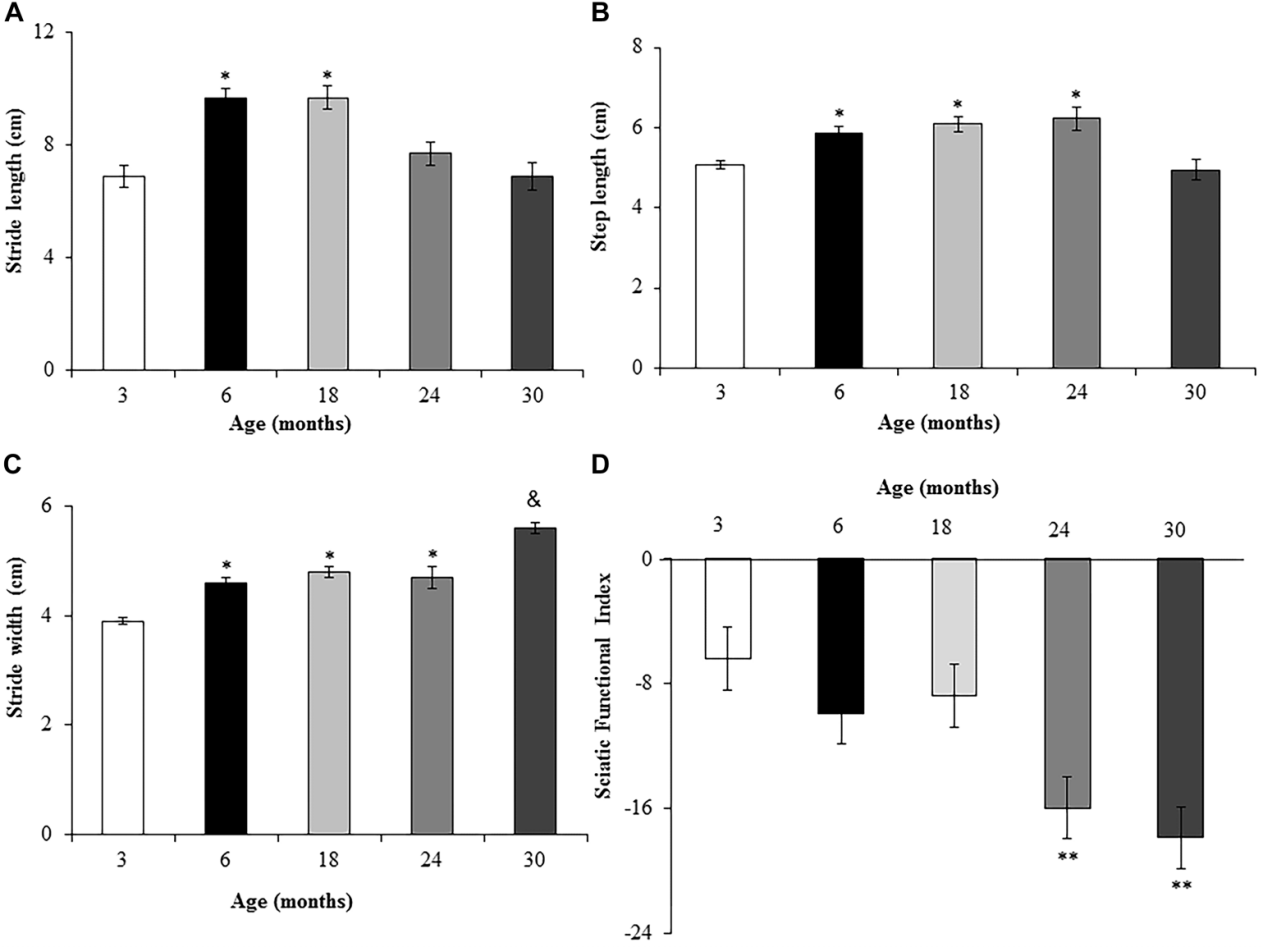
Assessment of stride length (**A**), step length (**B**), stride width (**C**), and sciatic functional index (**D**) of Wistar rats of different ages. Data are reported as means±SE. *P<0.05 compared to 3-month-old rats; **P<0.05 compared to 3-, 6-, and 18-month-old rats; ^&^P<0.05 compared to all groups (P<0.05, one-way ANOVA followed by Tukey's *post hoc* test).

The assessed gait parameters differed among ages. Stride length was significantly greater (43%) for 6- and 18-month-old rats compared to 3-month-old rats (Figure 4A), but there was no significant difference between the former. Stride length also did not differ significantly between 3-month-old rats and 24- and 30-month-old rats. Interestingly, 30-month-old rats had a stride length similar to that of 3-month-old rats. Stride length was 10% lower at 30 months than at 24 months. Step length was significantly greater for 6-, 18-, and 24-month-old rats compared to 3-month-old rats (Figure 4B). Interestingly, step length was similar for 3- and 30-month-old rats. Stride width was 25% greater for 6-, 18-, and 24-month-old rats, and 50% greater for 30-month-old rats, compared to 3-month-old rats. Stride width was 20% greater for 30-month-old rats compared to 6-, 18-, and 24-month-old rats (Figure 4C).

**Figure 5.**
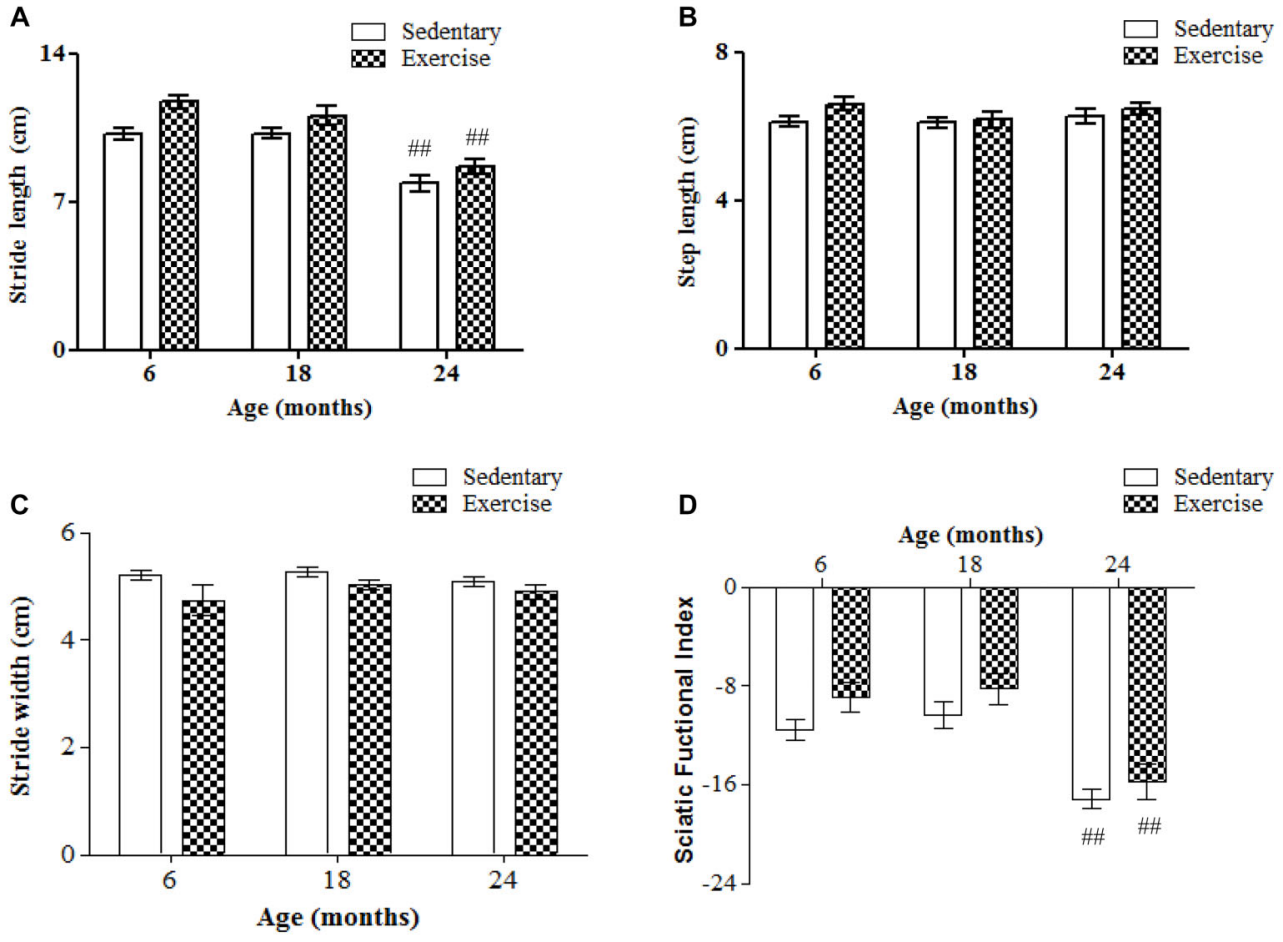
Assessment of stride length (**A**), step length (**B**), stride width (**C**), and sciatic functional index (**D**) of Wistar rats of different ages after 12 weeks of treadmill running. Data are reported as means±SE. ^##^P<0.05 compared to 6- and 18-month-old rats (two-way ANOVA followed by Tukey's *post hoc* test).

The SFI differed among ages. Values of SFI did not differ significantly among 3-, 6-, and 18-month-old rats (Figure 4D), with the values being near zero, indicating normal sciatic nerve function. However, while SFI values were around –6 for 3-month-old rats, the values were approximately –9.5 for 6- and 18-month-old rats, indicating a decrease of about 48% for 6- and 18-month-old rats compared to 3-month-old rats. The SFI was significantly lower for 24- and 30-month-old rats (150 and 180%, respectively), compared to 3-month-old rats. The SFI was also significantly lower for 24- and 30-month-old rats (68 and 89%, respectively) compared to 6-month-old rats. None of the gait parameters exhibited significant changes after low-intensity exercise (Figure 5).

**Figure 6.**
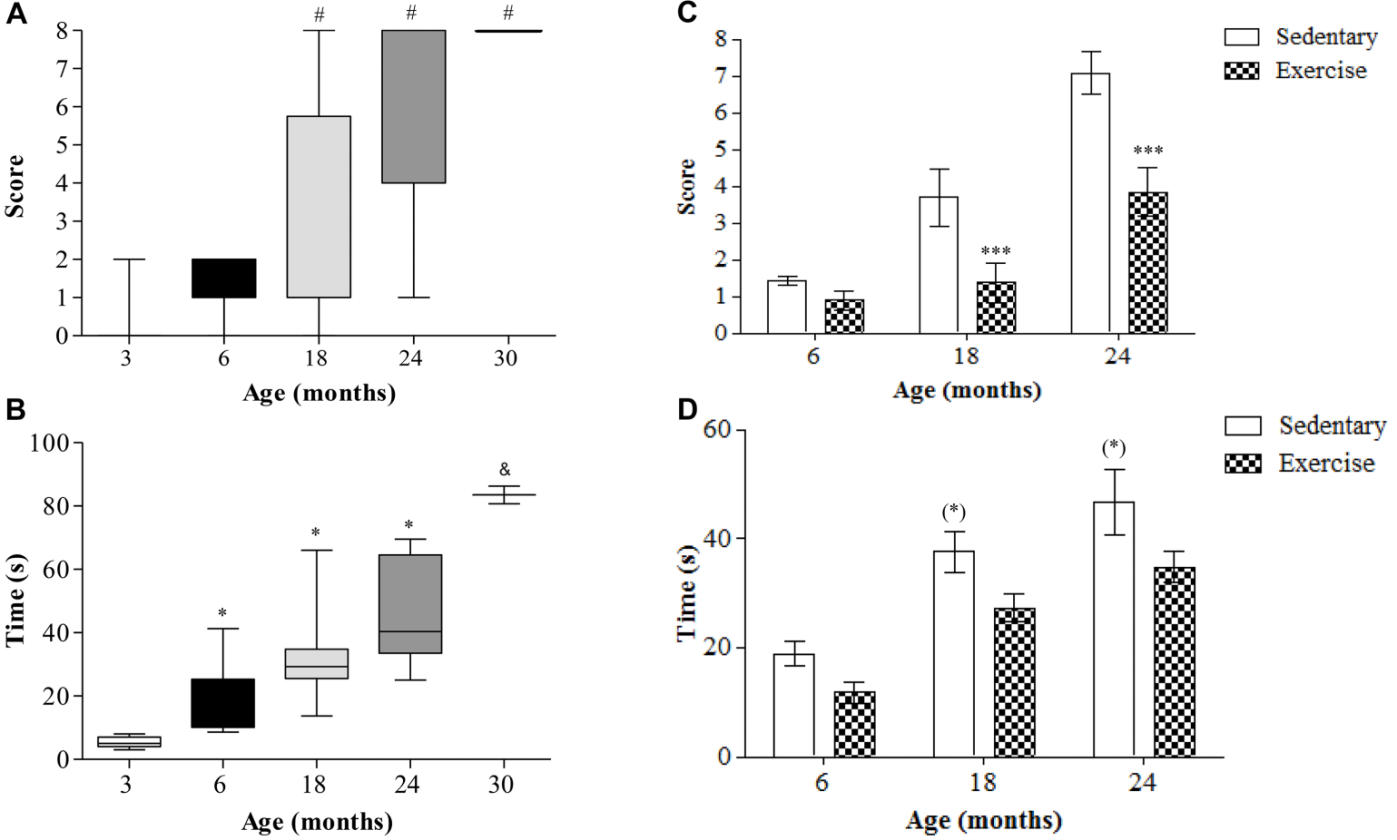
Responses to balance beam test in terms of total score (**A**,**C**) and total time to traverse the beam (**B**,**D**) for Wistar rats of different ages (**A**,**B**) and after 12 weeks of treadmill running (**C**,**D**). Data are reported as medians and 10–90% interquartile ranges (**A**,**B**) and means±SE (**C**,**D**). ^#^P<0.05 compared to 3- and 6-month-old rats; ***P<0.05 compared to sedentary rats over the same age; *P<0.05 compared to 3-month-old rats; ^(*)^P<0.05 compared to 6-month-old rats; ^&^P<0.05 compared to all groups (**A** and **B**, Kruskal-Wallis test followed by Dunn's *post hoc* test and **C** and **D**, two-way ANOVA followed Tukey's *post hoc* test).

Balance beam test scores differed among ages. Three-month-old rats had scores ranging between 0 and 2, but with a median of 0, indicating that these rats had few foot slips from the beam. The scores for 6-month-old rats ranged between 1 and 2 (Figure 6A), while those for 18-month-old rats ranged widely, but with the median being mainly between 1 and 4. The 24- and 30-month-old rats had obvious problems traversing the beam, with scores for 24-month-old rats ranging between 5 and 8 while 30-month-old rats had a score of 8.

Time to traverse the beam differed among ages (Figure 6B). While the average time taken by 3-month-old rats to traverse the beam ranged between 5 and 7 s, the time for 6- and 18-month-old rats ranged from 10–20 s and 10–60 s, respectively. The median time for 24-month-old rats ranged between 30 and 60 s, while all 30-month-old rats failed to traverse the beam but were able to reach an average time of 90 s with the assistance of a researcher.

Regular low-intensity exercise significantly ameliorated the scores for rats in the balance beam test. After exercise, 6-month-old rats had scores between 0 and 1, which indicated an amelioration of 36% in this parameter compared to sedentary rats of the same age. The scores were 1 and 2 for 18-month-old rats and 1 and 6 for 24-month-old rats, which revealed an amelioration of 62 and 47%, respectively, compared to sedentary rats of the same age (Figure 6C). Time to traverse the beam did not differ significantly after exercise (Figure 6D).

### Antioxidant parameters

**Figure 7.**
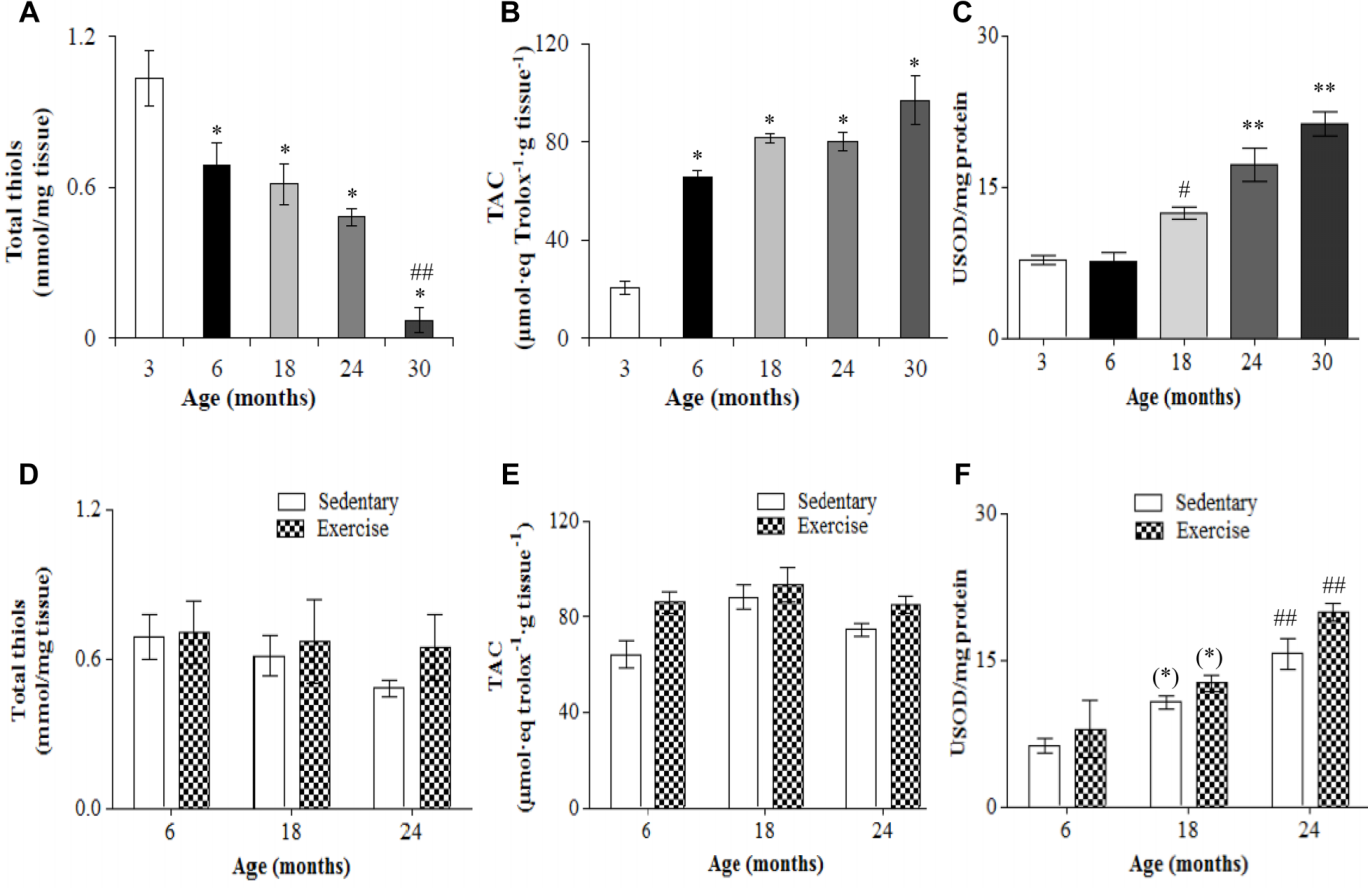
Total thiol content (**A**,**D**), total antioxidant capacity (TAC) (**B**,**E**), and superoxide dismutase (USOD) activity (**C**,**F**) in the lumbosacral spinal cord of Wistar rats of different ages (**A**–**C**) and after 12 weeks of treadmill running (**D**–**F**). Data are reported as mean±SE. *P<0.05 compared to 3-month-old rats; ^#^P<0.05 compared to 3- and 6-month-old rats; ^##^P<0.05 compared to 6- and 18-month-old rats; **P<0.05 compared to 3-, 6-, and 18-month-old rats; ^(^*^)^P<0.05 compared to 6-month-old rats of the same experimental group (one-way (**A**–**C**) and two-way (**D**–**F**) ANOVA followed by Tukey's *post hoc* test).

Total thiol content of the spinal cord was lower for older ages (Figure 7A). Despite not being significant, total thiol content was about 33% lower in the spinal cord of 6-month-old rats compared to 3 months. Total thiol was also lower in the spinal cord of 18-month-old rats, although the difference (41%) was significant only compared to 3-month-old rats. Total thiol content was 12% lower at 18 months than at 6 months. Total thiol content was also significantly lower in 24- and 30-month-old rats compared to 3. Total thiol content was 53% lower for 24-month-old rats compared to 3, and 21% lower compared to 18. The 30-month-old rats had lower total thiol content than 24-month-old rats, being approximately 93, 90, 88, and 85% lower compared to 3-, 6-, 18-, and 24-month-old rats, respectively. Total antioxidant capacity was low in the spinal cord of 3-month-old rats, and higher at 6, 18, 24, and 30 months (Figure 7B). The capacity at 6 months was 225% higher than that at 3 months, while at 18 and 24 months it was approximately 300% higher, and at 30 months 385% higher. The TAC was approximately 23% higher at 18 and 24 months compared to 6 months, and around 49% higher at 30 months. The TAC at 30 months was 21% higher than that at 24 months. SOD activity did not differ significantly between 3 and 6 months, but was higher at 18, 24, and 30 months compared to 3 months (Figure 7C), being 60, 115, and 165% higher, respectively. Interestingly, regular low-intensity exercise did not significantly change antioxidant parameters in the spinal cord of 6-, 18-, and 24-month-old rats (Figure 7D–F).

### Pro-oxidant parameters

**Figure 8.**
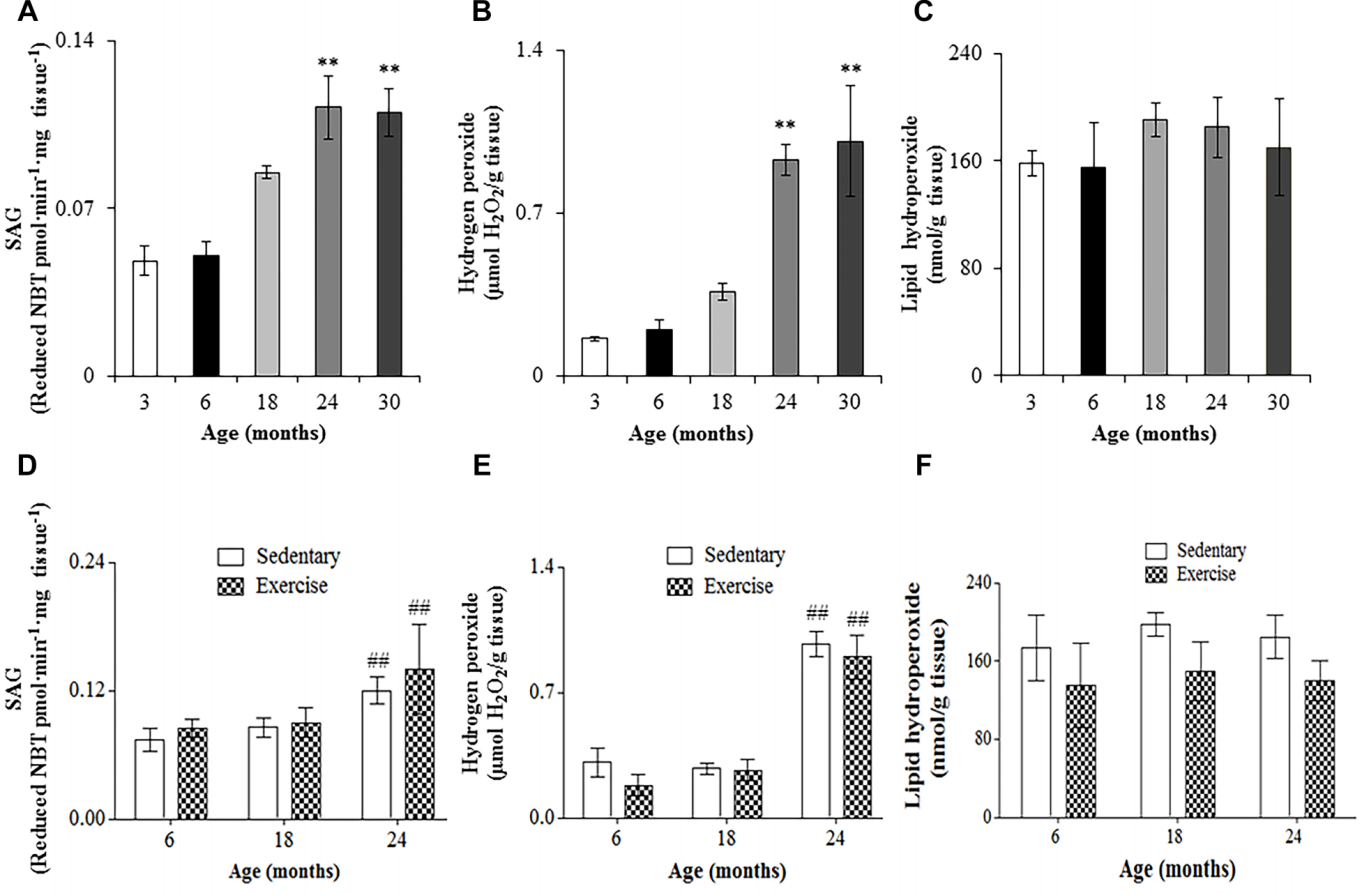
Superoxide anion generation (SAG) (**A**,**D**), hydrogen peroxide (**B**,**E**), and lipid hydroperoxide (**C**,**F**) levels in the lumbosacral spinal cord of Wistar rats of different ages (**A**–**C**) and after 12 weeks of treadmill running (**D**–**F**). Data are reported as mean±SE. **P<0.05 compared to 3-, 6-, and 18-month-old rats; ^##^P<0.05 compared to 6- and 18-month-old rats (one-way (**A**–**C**) and two-way (**D**–**F**) ANOVA followed by Tukey's *post hoc* test).

Pro-oxidant parameters differed among ages. The level of SAG was similar in the spinal cord of 3- and 6-month-old rats (Figure 8A). Despite not being significant, the level of SAG was 73% greater in the spinal cord of 18-month-old rats. This parameter increased significantly in the spinal cord at 24 and 30 months, being approximately 124% compared to 3 months, but 29% compared to 18 months. Levels of H_2_O_2_ were similar in the spinal cord at 3 and 6 months. This parameter was 125% greater in the spinal cord at 18 months, but not significantly so compared to 3 and 6 months (Figure 8B). Levels of H_2_O_2_ were similar in the spinal cord of 24- and 30-month-old rats; however, at 24 and 30 months, rats had higher levels of H_2_O_2_ (approximately 494%) compared to 3 months. The 24- and 30-month-old rats had approximately 164% greater H_2_O_2_ than 18-month-old rats. No significant differences were found among 3-, 6-, 18-, 24-, and 30-month-old rats for lipid hydroperoxides (Figure 8C). Exercise did not induce significant changes in pro-oxidant parameters (Figure 8D–F).

## Discussion

The present study assessed metabolic parameters, motor coordination and equilibrium, and spinal cord oxidative biomarkers in rats of different ages. The lack of changes in body weight, metabolic markers, and soleus muscle weight/body weight ratio with age was in line with previous studies (13,27,28). The slight decrease in blood glucose levels for old rats may be related to senescence; the median survival age for Wistar rats is 29–30 months (13). The maintenance of triglyceride levels during aging may be associated with diet. Rats were fed a standard diet throughout the entire experimental period, including prior to death. Fed aged rats did not experience changes in blood triglyceride levels (28). Thus, rats of the present study experienced changes that are commonly found during aging.

The age-induced changes in the balance-beam test and stride length were in line with previous research (13); however, those authors did not use 3-month-old rats. Thus, the results presented here add new information on these parameters in young rats. Interestingly, stride length was similar for 3- and 30-month-old rats. Recent research found longer stride lengths for middle-age rats than for 3-month-old rats (29), with the stride length from young rats similar to that found in the present study. This result reinforces our measurements for all rats and shows that stride length returns to values similar to that found in young adult rats.

As with stride length, step length increased from young adult rats to middle age rats, but then decreased in old age. Interestingly, the step length found for 30-month-old rats was similar to that found for 3-month-old rats. Thus, stride and step length returned to values found at 3 months in spite of the difference in body size and weight.

In contrast, stride width increased with advancing age, with old rats having the greatest stride width. Since a stable gait requires control of the position of the center of mass of the body relative to the base of support (30), greater stride width may be a compensatory strategy to keep the center of mass of the body within the base of support in old rats.

The present study is the first to demonstrate that SFI changes with age. This index provides a very precise and reliable measure of the sciatic nerve condition and is quickly and easily applied to large populations (16). These authors reported an SFI near zero for normal young rats. We also found SFI values near zero for 3-, 6-, and 18-month-old-rats, but lower values for 24- and 30-month-old rats. Since SFI is very sensitive to lesion in this nerve (16), the decreased SFI found in 24- and 30-month-old rats in the present study indicates sciatic nerve lesion. In fact, morphological changes were found in peripheral nerves of 10- and 24-month-old Wistar rats (31).

Parallel to behavioral changes, the levels of SAG and H_2_O_2_ increased in the spinal cord of aged rats. These results are in line with research showing increased ROS generation in the spinal cord with advancing age (4). However, our study is the first to demonstrate that the increase is associated with high SAG and H_2_O_2_. Since high levels of ROS exceed the adaptive tolerance of cells, resulting in significant oxidative damage, apoptosis, and necrosis (3), it is possible that the observed increase in SAG and H_2_O_2_ may be related to the changes found in gait and balance with advancing age. The spinal cord is a vital connector and integrator, carrying and manipulating efferent motor and afferent sensory information between the brain and the periphery to control a multitude of physiological process, including locomotion and balance (30). Since elevated levels of SAG and H_2_O_2_ were found in the spinal cord of the same rats that exhibited poor performance in the balance beam test and changes in the gait parameters, a relationship between these results cannot be discarded. However, it is impossible to address direct or indirect relationships between ROS and behavioral responses. Cell failure can be caused by ROS through interference with signaling pathways and their associated components, in addition to imposing direct damage, thus incorporating both the beneficial and malign nature of ROS (3). Thus, a relationship between SAG and H_2_O_2_ and deficits in behavioral responses of aged rats is possible, although further studies are necessary to better understand this relationship.

Interestingly, SAG and H_2_O_2_ levels were similar in the spinal cord of 24- and 30-month-old rats. This similarity may be related to age-induced functional changes. Previous research showed elevated H_2_O_2_ production in brain mitochondria of 24-month-old rats (32). According to these authors, the content of mitochondrial cytochrome c (terminal enzyme complex of the inner mitochondria membrane electron transport chain that catalyzes electron transfer from reduced cytochrome c to molecular oxygen) remains constant in 24-month-old rats, although its functions are impaired. In this context, it can be suggested that the lack of significant changes in SAG and H_2_O_2_ may be related to maintenance of enzymatic activity parallel to its functional deficit in 30-month-old rats. However, it is also necessary to consider that elevated SAG and H_2_O_2_ may be associated with age-related neurochemical changes. Pronounced alterations in neurotransmitter markers have been demonstrated in aged nervous tissue, with the spinal cord being more severely affected than most cerebral regions in 30-month-old rats (33). Free radicals are generated in cells under oxidative stress and when signaling pathways are activated (5).

Total thiol content gradually decreased in the spinal cord with advancing age, with the greatest decrease being in old rats. This result is similar to that found in 24–26-month-old Wistar rats (7). Thus, our results reinforce that thiol content decreases with age. In addition, the present study adds new evidence since it revealed a decrease in total thiol content for ages of 18 and 30 months, which was particularly dramatic for the latter. Decreased total thiol content may be indicative of reduced efficiency of S-thiolation as a mechanism of antioxidant defense during aging, thus creating an increased risk of irreversible oxidation of -SH groups of proteins (7). In this context, our results indicate that the irreversible oxidation of -SH groups of proteins occurs during aging, but is increased in the spinal cord of old rats. Protein oxidation modifies several amino acids and protein aggregation and fragmentation (7). Thus, it can be suggested that these changes are particularly elevated in the spinal cord of old rats.

The decrease in total thiol content may be related to H_2_O_2_. Thiol oxidation occurs by a two-electron mechanism, most commonly involving H_2_O_2_ (5). According to that author, superoxide reacts with thiol but, at least for small molecules, the rate is slow and the reaction is not a direct electron transfer, but instead generates the thiyl radical, which regenerates superoxide and forms disulfide as the major product. This is a chain reaction but the chain is short, probably because superoxide reacts with intermediate radicals, and thiols are unlikely to compete with SOD (5). In fact, SOD activity was found to increase in the spinal cord with advancing age. Superoxide dismutase is the enzyme that converts superoxide radicals into H_2_O_2_ (3).

Despite a decrease in total thiol content, TAC increased in the spinal cords of 6-, 18-, 24-, and 30-month-old rats. This result may be related to changes in other antioxidant systems. TAC represents both the enzymatic and non-enzymatic antioxidant compounds in the body, such as SOD, catalase, glutathione peroxidase, and glutathione (34). In fact, SOD activity increased in old rats. The interrelated changes in pro-oxidant and antioxidant defenses may be related to the lack of significant changes in lipid hydroperoxide levels with advancing age.

The present study also demonstrated the effects of regular low-intensity exercise on metabolic, behavioral, and oxidative parameters during aging. Exercise did not induce significant changes in body weight, blood glucose, and triglycerides. These results are in line with previous research (35). Interestingly, low-intensity exercise did not reduce total cholesterol. This exercise program also did not prevent age-induced decrease in soleus muscle weight/body weight ratio. It has been demonstrated that rats experience reduced total cholesterol after 12 weeks of low-intensity exercise (36). Moderate-intensity continuous training had a protective effect on relative soleus weight in 18-month-old female Sprague Dawley rats (35). Since frequency and/or intensity of exercise differed between the studies, these factors may be related to the lack of changes in total cholesterol and soleus muscle weight/body weight ratio, as shown for high- and moderate-intensity exercise (37). However, further studies are necessary to clarify this hypothesis.

While low-intensity exercise did not change gait parameters, it ameliorated performance in the balance beam test. This test assesses simple (static) equilibrium (38). Walking requires complex motor unit re-innervation coordinated by cortically integrated sensory feedback (39). Thus, our results suggested that regular low-intensity exercise improved static equilibrium, but not complex motor coordination. Since locomotor features depend on postural status at each step, with and without perturbations (40), the improvement in static equilibrium should be contributing to prevent changes in gait. However, this appears to be insufficient to change SFI. It is probable that greater training duration and/or volume are necessary to change gait parameters.

Training duration and volume may also be responsible for the lack of significant changes in spinal cord oxidative biomarkers after low-intensity exercise. Recent research revealed that prolonged mild treadmill exercise (50% of maximal exercise capacity, 5 days/week, 40 min/day, during 10 weeks) significantly decreased H_2_O_2_ levels in the spinal cord of aged Lewis rats (9). However, it must be taken into account that our study had a small number of rats per group. This imposes a strong limitation to the study, which weakened comparisons between groups and could result in false-negative findings. The statistical comparison in our study was always borderline. Thus, we are uncertain whether low-intensity exercise induced, or not, changes in gait and spinal cord oxidative biomarkers in aged rats, which is in need of further evaluation. In particular, it is necessary to increase the number of rats per group. It is also necessary to compare the effects of high- and low-intensity exercise on gait and spinal cord oxidative parameters to establish the intensity of exercise needed to prevent age-induced changes in these parameters. Thus, we cannot rule out a beneficial effect of low-intensity exercise on gait and spinal cord oxidative biomarkers.

In conclusion, the rats of the present study experienced changes in gait, balance, and spinal cord oxidative biomarkers during aging. Regular low-intensity exercise significantly improved performance in the balance beam test. Since the present study possessed limitations (small number of rats per group), a beneficial effect of low-intensity exercise on gait and spinal cord oxidative biomarkers cannot be ruled out.
